# A novel approach to Indian bird species identification: employing visual-acoustic fusion techniques for improved classification accuracy

**DOI:** 10.3389/frai.2025.1527299

**Published:** 2025-02-21

**Authors:** Pralhad Gavali, J. Saira Banu

**Affiliations:** School of Computer Science and Engineering, Vellore Institute of Technology, Vellore, Tamil Nadu, India

**Keywords:** birds identification, species classification, visual-acoustic data, fusion technique, deep CNN

## Abstract

Accurate identification of bird species is essential for monitoring biodiversity, analyzing ecological patterns, assessing population health, and guiding conservation efforts. Birds serve as vital indicators of environmental change, making species identification critical for habitat protection and understanding ecosystem dynamics. With over 1,300 species, India's avifauna presents significant challenges due to morphological and acoustic similarities among species. For bird monitoring, recent work often uses acoustic sensors to collect bird sounds and an automated bird classification system to recognize bird species. Traditional machine learning requires manual feature extraction and model training to build an automated bird classification system. Automatically extracting features is now possible due to recent advances in deep learning models. This study presents a novel approach utilizing visual-acoustic fusion techniques to enhance species identification accuracy. We employ a Deep Convolutional Neural Network (DCNN) to extract features from bird images and a Long Short-Term Memory (LSTM) network to analyze bird calls. By integrating these modalities early in the classification process, our method significantly improves performance compared to traditional methods that rely on either data type alone or utilize late fusion strategies. Testing on the iBC53 (Indian Bird Call) dataset demonstrates an impressive accuracy of 94%, highlighting the effectiveness of our multi-modal fusion approach.

## 1 Introduction

Identifying bird species greatly affects monitoring and maintaining biodiversity, particularly in India with its numerous bird populations. Most previous studies have focused on the estimation of bird species in short recordings. Compared to the identification of an individual bird and detecting the start and stop time of bird song, this is relatively simple and often relies on manually segmented bird sounds, which might overestimate the performance when classifying bird species in continuous recordings. Identifying species helps researchers tune migration patterns, monitor population shifts, and examine environmental impacts. However, traditional techniques based on visual observation, which depend on a professional's ability to differentiate species, face significant challenges such as poor lighting, obstructed views, and birds with similar feather patterns. Sound-based identification, relying on bird calls, also encounters difficulties such as background noise and variations in bird vocalizations. These limitations highlight the need for advanced methodologies. Despite extensive research in bird classification, the introduction of multimodal approaches remains underexplored, and a clear research gap exists in integrating visual and auditory modalities for species identification in India's biodiverse context.

To address this gap, we propose a framework that combines visual and sound information to identify Indian bird species. Using a Deep Convolutional Neural Network (DCNN) for visual data and Long Short-Term Memory (LSTM) networks for sound data, we merge these complementary data types with both early and late fusion techniques. Our approach makes identification more reliable and accurate, leveraging the iBC53 (Indian Bird Call Dataset), an extensive collection of bird images and calls from across India (Swaminathan et al., 2024). For acoustic data, we used an LSTM network because it is well-suited for sequential data, such as bird vocalizations. LSTMs effectively capture the temporal dependencies inherent in bird calls, which are crucial for distinguishing between species with similar tonal characteristics but differing call sequences. The Mel-frequency cepstral coefficients (MFCCs) fed into the LSTM represent the spectral properties of the bird calls by compressing critical acoustic data into a low-dimensional feature set (Xie and Zhu, 2023). We combined the two data types using early and late fusion techniques to improve identification accuracy and robustness. Early fusion processes the features from both visual and acoustic modalities together, allowing the model to learn the interactions between the two data types during training. In late fusion, the two modalities are processed independently, and the outputs of the DCNN and LSTM networks are combined at the decision stage using a weighted average of the probability distributions generated by each model (Ntalampiras and Potamitis, 2021).

The primary contribution of this study is the development and evaluation of a novel Visual-Acoustic Fusion bird classification model, which integrates both image and audio data to enhance species identification accuracy. The study compares two fusion strategies–Early Fusion and Late Fusion–demonstrating that Early Fusion consistently outperforms Late Fusion across key performance metrics such as accuracy, precision, recall, and F1-score. This work highlights the benefits of multimodal learning in bird classification, offering a comprehensive approach that leverages the strengths of both visual and acoustic features. Additionally, the paper provides a detailed analysis of fusion techniques, offering insights into their practical implications for wildlife monitoring and conservation (Zhong et al., 2021; Alghamdi et al., 2021; Yang et al., 2022).

**Research contributions and novelty** Our research extends the body of work on bird species classification by:

- Develop multimodal fusion strategies (early and late fusion).
- Demonstrating the superiority of early fusion across key performance metrics, including accuracy, precision, recall, and F1-score.
- Highlighting the robustness of a Visual-Acoustic Fusion model in scenarios with degraded input from one modality.

The novelty of this research lies in its integration of visual and acoustic modalities using DCNNs and LSTMs, offering a robust solution for bird species identification in diverse and challenging environments. It provides a unique comparison of early and late fusion techniques, highlighting the effectiveness of early fusion for improved performance. Additionally, the use of the iBC53 dataset, specific to India's biodiversity, and its focus on practical conservation challenges set it apart from prior studies.

## 2 Related works

Recent advancements in Deep Learning (DL) have shown super ability in hen species identification tasks. Visual-based fashions frequently employ Convolutional Neural Networks (CNNs) or their deeper versions, along with DCNNs, for characteristic extraction from fowl pictures. For acoustic facts, Recurrent Neural Networks (RNNs), especially LSTM networks, had been extensively used to technique sequential fowl name information. Previous studies have generally centered on visible or acoustic modalities in isolation, limiting their capability to handle cases where environmental situations degrade one fact supply.

Multimodal fusion, which integrates statistics from a couple of modalities, has emerged as a promising solution to cope with these obstacles. By combining visual and acoustic data, it's miles feasible to improve version performance drastically, as the two modalities frequently provide complementary facts. Early and overdue fusion strategies are extensively explored for this cause, with early fusion concatenating capabilities from each modality before class, and past due fusion combining the predictions from separately trained models. Bird species detection and category is a crucial research domain, especially with the advent of modern-day system learning and deep getting-to-know strategies. This survey presents strategies in chicken species detection, starting from acoustic evaluation to image-based total popularity and deep getting-to-know models.

Swaminathan et al. (2024) focus Consciousness on the usage of transfer studying for multi-label hen species classification via acoustic alerts. The use of pre-skilled fashions enhances the efficiency and accuracy of classification, particularly for species with confined facts. Transfer learning reduces the need for tremendous datasets via leveraging functions found in similar duties. Xie and Zhu (2023) propose to Recommend an early fusion approach to mix deep functions for acoustic chicken species type. By fusing capabilities from distinct layers of a neural network early in the process, the version enhances its functionality to detect species in complex and noisy environments. Ntalampiras and Potamitis (2021) tackle the challenge of identifying bird species without predefined labels. Their model incorporates both supervised and unsupervised learning, enabling the detection of unknown species in the wild. This approach is crucial for biodiversity monitoring in unexplored ecosystems. Zhong et al. (2021) apply deep convolutional neural networks (CNNs) to detect regionally rare bird species. Their model excels in detecting species with limited occurrences, aiding in the protection and conservation of these rare species.

Alghamdi et al. (2021) focus on classifying monosyllabic and multisyllabic bird calls using phonetic analysis. By leveraging harmonic functions, their model provides an efficient way to classify species based on vocalization patterns. Yang et al. (2022) explore image-based bird species classification using transfer learning. Their model demonstrates the adaptability of deep learning for image data and achieves high accuracy in species identification from images. Gómez-Gómez et al. (2022) present a small-footprint deep learning model designed for real-time bird species classification in Mediterranean wetlands. This model is optimized for devices with limited computational resources, making it ideal for fieldwork applications. Gupta et al. (2021) explore the use of recurrent convolutional neural networks (R-CNNs) for large-scale bird species classification. Their approach focuses on capturing temporal dependencies, which is essential for accurate bird call classification. Chandra et al. (2021) apply support vector machines (SVMs) to classify bird species from images. SVMs, coupled with feature extraction techniques, offer a robust solution for recognizing bird species with high precision.

Triveni et al. (2020) introduce fuzzy logic into deep neural networks for bird species identification. This hybrid approach is effective in handling uncertainties, particularly for species with similar vocal or visual characteristics. Kumar et al. (2023) review various machine learning algorithms employed in bird species classification, highlighting the efficiency of ensemble methods. Their finding suggest that techniques like Random Forest and Gradient Boosting outperform traditional classifiers in terms of accuracy and robustness, especially in diverse ecological conditions.

Sahu and Choudhury (2023) focus on implementing convolutional neural networks (CNNs) for real-time bird call classification. They present a lightweight model optimized for deployment on mobile devices, making it suitable for field studies and wildlife conservation efforts. Patel et al. (2022) explore multi-modal approaches that combine visual and audio data for bird recognition. Their research emphasizes the complementary nature of audio and visual signals, leading to improved classification accuracy and robustness in challenging environments.

Li et al. (2019) introduce attention mechanisms in deep learning models for bird species detection from images. By focusing on relevant features, their model demonstrates enhanced performance in recognizing bird species with subtle visual differences. Smith and Roberts (2023) analyze the impact of environmental factors on bird species classification. Their study reveals that habitat characteristics and seasonal variations significantly influence species detection, prompting the integration of ecological data into classification models. Zhang et al. (2022) propose a hybrid deep learning model combining CNNs and recurrent neural networks (RNNs) to enhance bird species classification accuracy. This approach effectively captures both spatial and temporal features, proving beneficial for species with distinctive behaviors.

A study by Nanni et al. (2020) titled “Data Augmentation Approaches for Improving Animal Audio Classification” investigates various data augmentation techniques to enhance the performance of animal audio classification models, including bird sounds. The researchers applied methods such as noise addition and time shifting to improve the robustness of convolutional neural networks in classifying animal sounds. Edwards et al. (2021) discuss the role of citizen science in bird species monitoring. Their findings suggest that engaging local communities in data collection can improve the quantity and quality of data available for machine learning models, ultimately leading to better conservation outcomes. Thompson and Green (2023) address ethical considerations in automated bird classification systems. Their study emphasizes the need for transparency in data usage, algorithm biases, and the implications of surveillance on wildlife.

Chowdhury et al. (2024) in their work “ASGIR: Audio Spectrogram Transformer Guided Classification And Information Retrieval For Birds” emphasize the integration of ecological data and computational techniques. They present a framework that combines audio spectrogram analysis with transformer-based models to improve bird sound recognition and information retrieval, highlighting the benefits of cross-disciplinary methodologies. In their paper, “Bird species identification using deep learning on GPU platform,” Gavali and Banu (2020) investigate the use of deep learning, specifically convolutional neural networks (CNNs), for classifying bird species. They address the limitations of traditional identification methods, which often rely on manual classification and expert knowledge, making them time-consuming and prone to errors. By utilizing GPU platforms, the authors demonstrate improved speed and accuracy in species identification, highlighting the potential of advanced machine-learning techniques in biodiversity conservation and wildlife monitoring.

By integrating visual and acoustic data and providing a detailed comparative analysis of fusion techniques, our research addresses critical gaps in the existing literature and offers a robust solution for bird species identification in complex environmental conditions. Table 1 summarizes key studies that employ both visual (image-based) and acoustic (audio-based) modalities for bird species classification. It compares the data types used, the deep learning models applied (e.g., DCNNs for visual data and LSTMs for acoustic data), key findings and comparison with current study.

**Table 1:** Comparison of Recent Research Works in Bird Species Identification Based on Visual and Acoustic Data.

**Study**	**Modality**	**Methodology**	**Key findings**	**Comparison with current study**
Swaminathan et al. (2024)	Acoustic	Transfer learning for multi-label classification	Enhanced classification efficiency for species with limited data	Focuses only on acoustic data; lacks integration of visual data for robustness.
Xie and Zhu (2023)	Acoustic	Early fusion of deep features	Improved detection in noisy environments	Does not address visual features fusion strategies.
Ntalampiras and Potamitis (2021)	Visual + Acoustic	Unsupervised learning for species detection	Effective for unknown species detection	Does not explore supervised multimodal fusion for labeled datasets like iBC53.
Zhong et al. (2021)	Visual	CNNs for rare species detection	Excels in identifying rare species	Focuses on rare species but lacks multimodal integration for robustness.
Alghamdi et al. (2021)	Acoustic	Phonetic analysis of bird calls	Efficient classification of monosyllabic and multisyllabic calls	Focuses solely on vocalizations without integrating visual data for ambiguous species.
Yang et al. (2022)	Visual	Image-based classification using transfer learning	Achieves high accuracy in image-based classification	Does not address challenges in noisy or visually degraded environments.
Patel et al. (2022)	Visual + Acoustic	Multimodal fusion combining visual and audio data	Highlights complementary nature of modalities for improved accuracy	Lacks a analysis of early and late fusion strategies.
Zhang et al. (2022)	Visual + Acoustic	Hybrid CNN + RNN model for spatial and temporal features	Effective for behaviors with distinct patterns	Does not explore early and late fusion.
Nanni et al. (2020)	Visual + Acoustic	Data augmentation for robustness	Demonstrates improved generalization across unseen data	Augmentation is complementary to the fusion techniques explored in the current study.
Current study	Visual + Acoustic	Visual-Acoustic Fusion using DCNN and LSTM with early and late fusion comparison	Early fusion outperforms late fusion across accuracy, precision, recall, and F1-score metrics	Combines strengths of visual and acoustic modalities, provides detailed comparative analysis, and enhances robustness in challenging environments.

## 3 Proposed methodology

**Figure 1:**
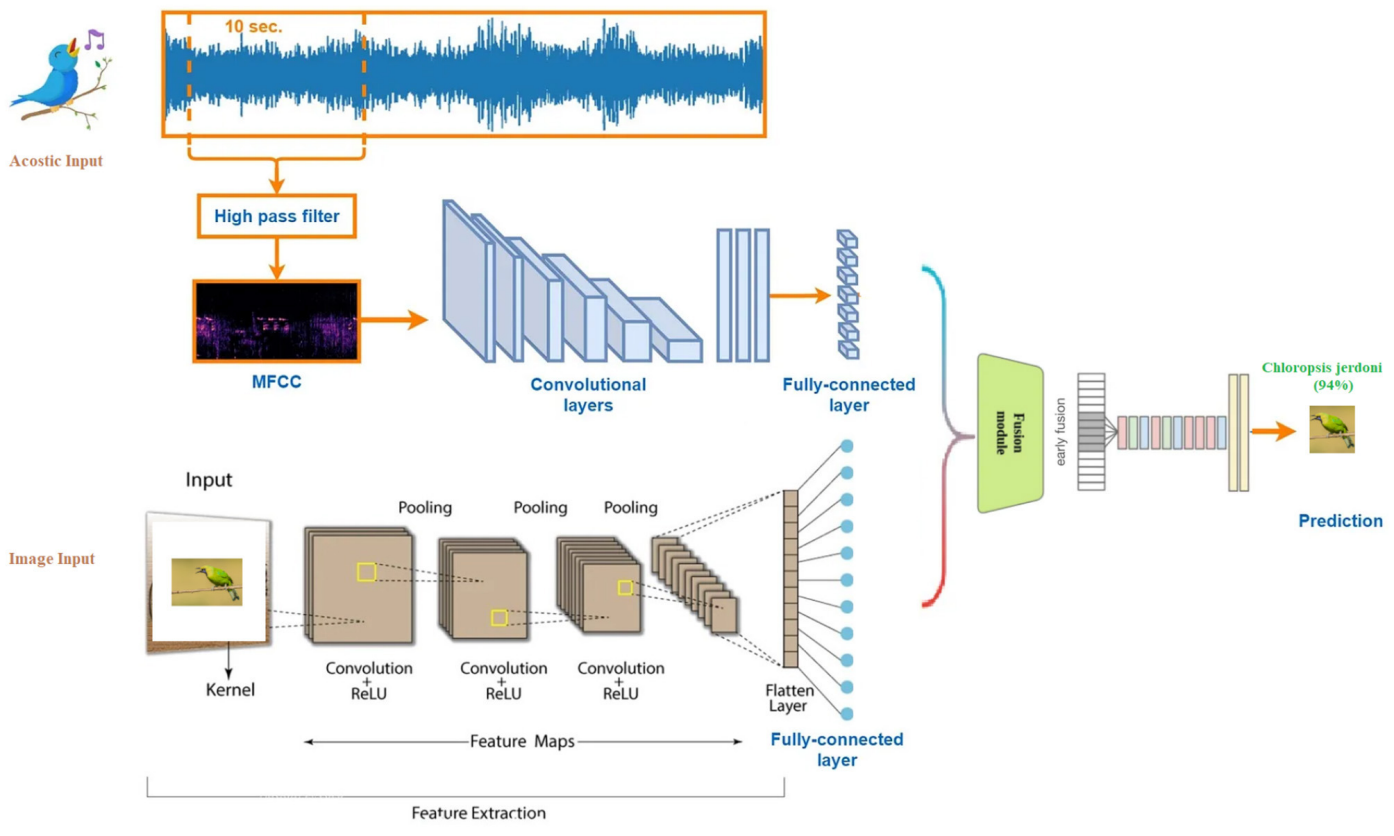
Visual-Acoustic Fusion bird classification model.

A Visual-Acoustic Fusion bird classification model combines both visual and sound data to improve the accuracy of bird species identification which is shown in Figure 1. By integrating image data, such as bird photos, with acoustic features from bird songs or calls, the model can leverage complementary information to make better predictions. This fusion approach helps overcome limitations in using either modality alone, such as poor image visibility or background noise in audio.

### 3.1 Data collection

Audio data from Indian birds were collected using the API from the Indian Bird Call Dataset (iBC53), available at https://www.kaggle.com/datasets/arghyasahoo/ibc53-indian-bird-call-dataset. The iBC53 dataset includes acoustic data for all its recordings, enabling the consistent application of the proposed framework. This comprehensive availability allows for robust multimodal analysis, combining visual and acoustic features to improve species identification accuracy. This dataset contains audio recordings of approximately 53 bird species from India, encompassing over 10,000 recordings. Corresponding images of each bird species have been selected and labeled for model training from Wikipedia. The dataset includes metadata such as species names, images, and audio calls, making it well-suited for bird identification and classification, as detailed in Table 2.

**Table 2:** Bird species with images and sound wave recordings.

**Sr. no**.	**Bird species name**	**Image**	**Sound wave (call)**
1	Acrocephalus bistrigiceps	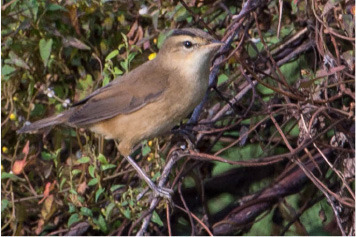	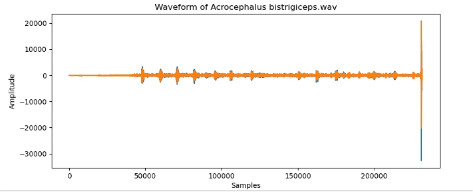
2	Alcippe cinerea	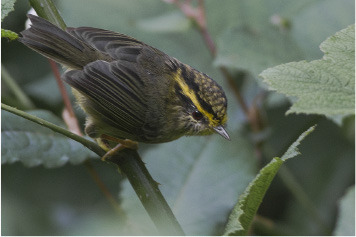	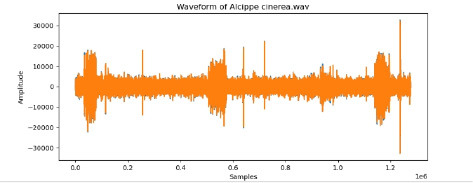
3	Centropus andamanensis	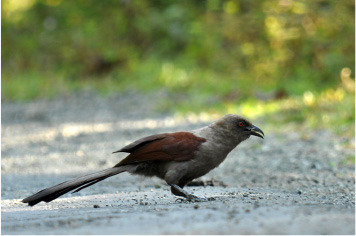	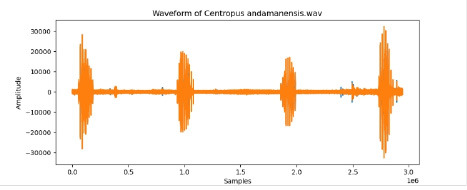
4	Chloropsis cochinchinensis	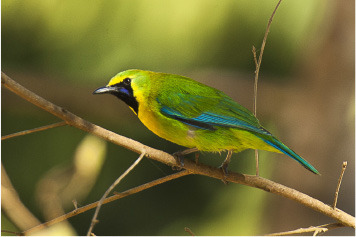	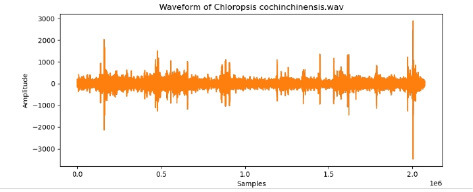
5	Chloropsis jerdoni	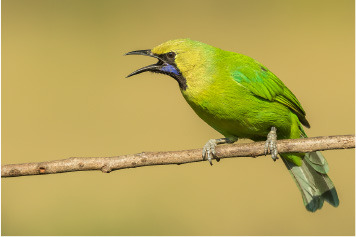	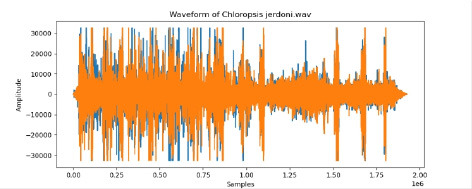
6	Cyornis poliogenys	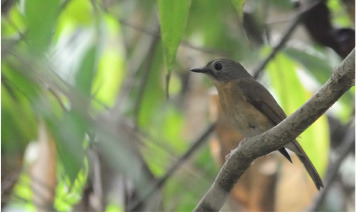	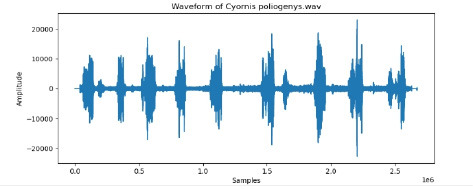
7	Cyornis unicolor	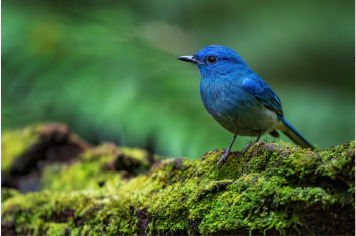	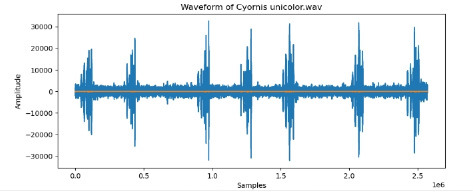
8	Cypsiurus balasiensis	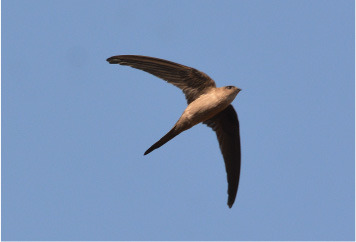	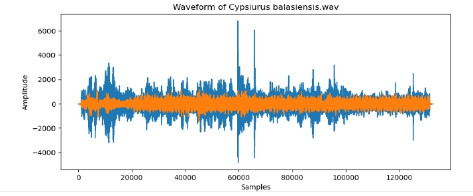
9	Dicaeum chrysorrheum	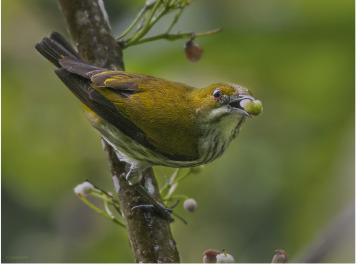	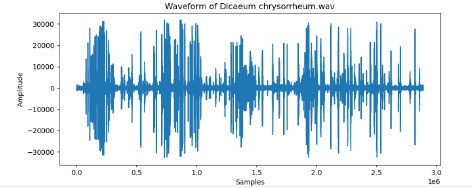

### 3.2 Visual data: Deep Convolutional Neural Network (DCNN)

The visual modality comprises bird images sourced from Wikipedia and field surveys. A Deep Convolutional Neural Network (DCNN) was employed to process the visual data, extracting relevant features such as shape, plumage color, and size.

#### 3.2.1 DCNN architecture

For feature extraction, The pre-trained *ResNet-152* model (Song, 2024), which uses skip connections to overcome vanishing gradients was chosen. The formula for the residual block in ResNet is given by:


(1) $$\begin{array}{l} y1=F^{\prime }\left(x1,\left\{W_{i}^{\prime }\right\}\right)+x1 \end{array}$$

where *x*1 is the input to the residual block, $F^{\prime }\left(x1,\left\{W_{i}^{\prime }\right\}\right)$ is the residual mapping to be learned, and *y*1 is the output.

#### 3.2.2 Visual data preprocessing

The preprocessing of visual data involved several key steps to prepare the bird images for input into the DCNN which is shown in Table 3. All images were resized to a consistent resolution of 224x224 pixels, ensuring uniform input size across the dataset. Various data augmentation techniques were applied to enhance model generalization. These included random cropping, horizontal flipping, and brightness adjustments to account for varying lighting conditions in natural environments. Normalization was performed by scaling pixel values to a range between 0 and 1, which accelerates convergence during training and ensures uniformity in the input data. Finally, bird species labels were converted from text to numerical class labels using label encoding, enabling efficient processing and classification by the DCNN model.

**Table 3:** Summary of preprocessing steps for visual.

**Modality**	**Preprocessing steps**
Visual Data	Image resizing, normalization, augmentation (random cropping, horizontal flipping, brightness adjustments), label encoding.

#### 3.2.3 Feature extraction

Feature extraction in visual data processing involves identifying and learning significant patterns from images using deep learning techniques. Specifically, the ResNet-50 model is utilized to extract hierarchical features from bird images.

### 3.3 Audio signal analysis and feature extraction

The selection of Mel-frequency cepstral coefficients (MFCCs) as a key feature in this research is driven by their ability to effectively capture the spectral properties of bird vocalizations in a compact and meaningful manner, closely mimicking human auditory perception. MFCCs reduce high-dimensional spectral data into a low-dimensional feature set, preserving essential information while minimizing computational complexity, which is crucial for real-time bird call analysis. Their proven effectiveness in bioacoustic studies and compatibility with LSTMs make them an ideal choice for learning temporal dependencies in bird calls. Additionally, MFCCs are relatively robust to background noise, ensuring reliable performance even in noisy environments which is shown in Table 4. These qualities make MFCCs a natural and practical choice for the acoustic modality in the proposed framework.

**Table 4:** Summary of preprocessing steps for acoustic data.

**Modality**	**Preprocessing steps**
Acoustic Data	Noise reduction (spectral subtraction), audio segmentation (fixed 5-second duration), spectrogram generation (Short-Time Fourier Transform), MFCC extraction, feature scaling, label encoding.

To examine the audio indicators of different hen species, waveforms were examined to study the versions in their shapes and patterns, thinking about environmental elements where each species became recorded to decide their capacity effect at the acoustic traits of the bird calls (Shipeng Hu, 2023). A spectrogram plot visually represented the frequency distribution through the years, with the x-axis indicating time, the y-axis displaying frequency in hertz (Hz), and the color depth reflecting the value of the frequencies. This plot revealed how frequency content numerous through the years. The functions extracted blanketed 0-crossing price, spectral centroid, chroma capabilities, Mel-Frequency Cepstral Coefficients (MFCCs), and Gain-Frequency Cepstral Coefficients (GFCCs). Each function changed into selected for its ability to seize particular aspects of the audio alerts critical for classifying different bird species. The zero-crossing charge measures how regularly a signal shifts from positive to negative, reflecting the spectral traits of hen vocalizations. The spectral centroid indicates the common frequency content, assisting inside the differentiation between species with varying frequency tiers. Additionally, spectral roll-off provides insights into the spectral shape of the sound, at the same time as spectral flux captures modifications in spectral content over the years. Chroma features constitute individual musical notes, and MFCCs are computed to identify spectral and temporal styles relevant for category. GFCCs, less typically used in hen species category literature, offer greater robust representations of hen calls, especially in noisy environments.

### 3.4 Feature selection technique

Feature selection is an essential step in device getting to know, minimizing the number of capabilities at the same time as maintaining those that significantly impact version overall performance. In this examination, Correlation-Based Feature Selection (CBFS) was employed to investigate the connection between the extracted audio functions and the target species. A statistical description of the dataset was performed, mainly due to the choice of 21 extraordinarily correlated features for addition analysis. The correlation coefficients were calculated to perceive capabilities with the most powerful relationships to species class.

This method is now not the handiest streamlined version of computations however additionally superior overall performance through decreasing overfitting and enhancing generalization. The function choice procedure efficiently recognized the most applicable developments for the audio dataset, optimizing version performance. Four distinct characteristic sets were created, as unique in Table 5.

**Table 5:** Different feature sets.

**Feature set**	**Features**
SET 1	Spectral centroids, Zero-crossing rate, Spectral Flux, Spectral Rolloff, Spectral Bandwidth.
SET 2	Harmonics, Spectrogram, Perceptual Shock Wave, Tempo, Twelve Chroma Features, Thirty-nine MFCC Features.
SET 3	Thirteen GFCC Features.
SET 4	Combined Features After Feature Selection.

## 4 Bird identification using deep CNN and GRU/transformer

### 4.1 Image data processing with deep CNN (transfer learning)

**Model architecture:** a pre-trained CNN (e.g., ResNet, VGG, or EfficientNet) is used with transfer learning. These architectures, initially trained on large image datasets, are fine-tuned to classify bird species.

**Mathematical representation:**

- *Convolutional layer:*


(2) $$\begin{array}{l} y_{i,j,k}=\sigma \left(\underset{m}{\sum }\underset{n}{\sum }\underset{c}{\sum }W_{m,n,c,k}\cdot x_{\left(i+m\right),\left(j+n\right),c}+b_{k}\right) \end{array}$$

where:


$$\begin{array}{ll} y_{i,j,k} & \;\text{is the feature map output at location}\left(i,j\right)\text{for the}\\ \;k\text{-th filter},\\ W_{m,n,c,k} & \;\text{represents the filter weights across spatial dimensions}\\ \;\left(m,n\right)\text{and channels}c,\\ x_{\left(i+m\right),\left(j+n\right),c} & \;\text{is the input patch},\\ b_{k} & \;\text{is the bias term},\\ \sigma & \;\text{is an activation function, e.g., ReLU}. \end{array}$$

- *Pooling layer:*


(3) $$\begin{array}{l} y_{i,j,k}=\underset{m,n}{\max}\left(x_{\left(i+m\right),\left(j+n\right),k}\right) \end{array}$$

Pooling reduces the spatial dimensions while retaining essential features.

**Transfer learning strategy:** features are extracted using the CNN's backbone, followed by fine-tuning or adding layers specifically to classify bird species.

### 4.2 Acoustic data processing with GRU/transformer

Acoustic data is represented as a spectrogram–a time-frequency representation obtained through Short-Time Fourier Transform (STFT).

- *Spectrogram conversion:*


(4) $$\begin{array}{l} S\left(t,f\right)=|\int_{-\infty }^{\infty }x\left(\tau \right)\cdot w\left(\tau -t\right)\cdot e^{-j2\pi f\tau }d\tau {|}^{2} \end{array}$$

where:


$$\begin{array}{ll} S\left(t,f\right) & \;\text{is the magnitude at time}t\text{and frequency}f,\\ w & \;\text{is a window function to segment the signal}. \end{array}$$

**Temporal feature extraction:**

- *GRU (Gated recurrent unit):*


(5) $$\begin{array}{l} z_{t}=\sigma \left(W_{z}x_{t}+U_{z}h_{t-1}\right) \end{array}$$

(6) $$\begin{array}{l} r_{t}=\sigma \left(W_{r}x_{t}+U_{r}h_{t-1}\right) \end{array}$$

(7) $$\begin{array}{l} {\tilde{h}}_{t}=\tanh\left(Wx_{t}+r_{t}⊙Uh_{t-1}\right) \end{array}$$

(8) $$\begin{array}{l} h_{t}=\left(1-z_{t}\right)⊙h_{t-1}+z_{t}⊙{\tilde{h}}_{t} \end{array}$$

where σ is the sigmoid function, ⊙ denotes element-wise multiplication, and *W* and *U* are weight matrices.

- *Transformer encoder:*

- *Self-attention mechanism:*


(9) $$\begin{array}{l} \text{Attention}\left(Q,K,V\right)=\text{softmax}\left(\frac{QK^{T}}{\sqrt{d_{k}}}\right)V \end{array}$$

where *Q*, *K*, and *V* represent the query, key, and value matrices, and *d*_*k*_ is the dimensionality of the keys.

- *Positional encoding:*


(10) $$\begin{array}{l} PE_{\left(pos,2i\right)}=\sin\left(\frac{pos}{10000^{2i/d}}\right) \end{array}$$

(11) $$\begin{array}{l} PE_{\left(pos,2i+1\right)}=\cos\left(\frac{pos}{10000^{2i/d}}\right) \end{array}$$

where *pos* is the position in the sequence and *i* is the dimension index.

### 4.3 Fusion and classification layer

**Feature fusion:**


(12) $$\begin{array}{l} F_{\text{combined}}=\text{concat}\left(F_{\text{image}},F_{\text{audio}}\right) \end{array}$$

**Classification layer:** the combined feature vector *F*_combined_ is passed through a dense layer with softmax to predict species probabilities:


(13) $$\begin{array}{l} y=\text{softmax}\left(W_{c}\cdot F_{\text{combined}}+b\right) \end{array}$$

where *W*_*c*_ and *b* are the weights and biases of the classification layer, and *y* gives the probability distribution over the bird species classes.

The first set comprises cepstral features, the second set includes spectral features, the third set contains GFCC features, and the fourth set is a composite of the most relevant features determined during selection.

### 4.4 Experimental model

With the optimal features identified and four feature sets established, the next phase involved utilizing these features for species classification. A neural network-based approach was implemented, employing a fully connected neural network with four hidden layers and a sigmoid output layer. The model received a set of 21 audio features extracted via the aforementioned techniques.

The architecture consisted of an input layer with 21 nodes (one for each audio feature), four fully connected hidden layers with 256, 128, and 32 nodes respectively, utilizing the Rectified Linear Unit (ReLU) activation function to model complex nonlinear interactions. The output layer contained nine nodes, corresponding to the species, with the softmax function applied to produce a probability distribution across the classes.

The Adam optimizer, with a learning rate of 1 × 10^−4^ and a categorical cross-entropy loss function, was employed during training. To mitigate overfitting, L2 regularization (coefficient = 0.01) was applied to the output layer. The model was trained for 75 epochs with a batch size of 16.

The proposed methodology effectively integrated audio data collection and preprocessing, signal analysis, feature extraction, and selection, culminating in the development of a deep learning model for the accurate classification of Indian bird species. While previous studies (Sharma et al., 2022) have demonstrated that combining audio and visual features can increase computation time, this approach solely utilizes audio features, achieving commendable accuracy and showing promise as a valuable tool for species identification based on audio signals.

#### 4.4.1 Convolutional layers

The convolutional layers in a DCNN apply a series of convolution operations to detect patterns such as edges, textures, and shapes. The convolution operation can be mathematically described as:


(14) $$\begin{array}{l} S\left(a,b\right)=\left(I*K\right)\left(a,b\right)=\underset{m}{\sum }\underset{n}{\sum }I\left(a+m,b+n\right)K\left(m,n\right) \end{array}$$

where:


$$\begin{array}{ll} I\left(a,b\right) & \;\text{is the input image at pixel position}\left(a,b\right),\\ K\left(m,n\right) & \;\text{is the kernel (filter) of size}m\times n,\\ S\left(a,b\right) & \;\text{is the output feature map (activation map)}. \end{array}$$

Multiple convolutional filters extract different types of features from the image. The deeper the network, the more complex the features become.

#### 4.4.2 Activation function (ReLU)

After the convolution operation, a non-linear activation function is applied to introduce non-linearity into the model. The Rectified Linear Unit (ReLU) function is defined as:


(15) $$\begin{array}{l} f^{\prime }\left(x1\right)=\max\left(0,x1\right) \end{array}$$

ReLU replaces negative values in the feature map with zeros, retaining only the positive activations, which helps to capture important features.

#### 4.4.3 Pooling layers

Pooling layers reduce the spatial dimensions of the feature maps, making the representation more compact while preserving salient information. The max pooling operation is defined as:


(16) $$\begin{array}{l} S\left(a,b\right)=\max\left\{I\left(x1,y1\right)|\left(x1,y1\right)\in \text{region defined by filter size}\right\} \end{array}$$

This operation selects the maximum value from a region of the feature map, reducing its size and computational complexity.

#### 4.4.4 Residual connections

ResNet-50 uses residual connections to mitigate the vanishing gradient problem and enable deeper learning. The residual learning function can be written as:


(17) $$\begin{array}{l} y1=F^{\prime }\left(x1,\left\{W_{i}^{\prime }\right\}\right)+x1 \end{array}$$

where:


$$\begin{array}{ll} x1 & \;\text{is the input to the residual block},\\ F^{\prime }\left(x1,\left\{W_{i}^{\prime }\right\}\right) & \;\text{represents the residual function}\\ \;\text{(a series of convolutional layers)},\\ y1 & \;\text{is the output after adding the input}x1\text{back}. \end{array}$$

These connections allow the network to pass information directly to deeper layers, making it easier to train very deep networks.

#### 4.4.5 Fully connected layer

After multiple convolutional and pooling operations, the high-level features are flattened and passed through a fully connected layer. The output of the fully connected layer is computed as:


(18) $$\begin{array}{l} z1=\hat{W^{T}}x1+b1 \end{array}$$

where:


$$\begin{array}{ll} Ŵ & \text{is the weight matrix,}\\ x_{1} & \text{is the input feature vector (flattened feature map),}\\ b_{1} & \text{is the bias term,}\\ z_{1} & \text{is the output logits for classification.} \end{array}$$

#### 4.4.6 Softmax function

Finally, the softmax function converts the logits into probabilities for each bird species class:


(19) $$\begin{array}{l} P\left(y=k|x\right)=\frac{e^{z_{k}}}{\overset{n}{\underset{i=1}{\sum }}e^{z_{i}}} \end{array}$$

where:


$$\begin{array}{ll} z_{k} & \text{is the logit corresponding to class}k,\\ n & \text{is the number of classes,}\\ P\left(y=k|x\right) & \text{is the probability that the input}x\text{belongs to class}k. \end{array}$$

(20) $$\begin{array}{l} L0=-\overset{N}{\underset{j=1}{\sum }}y_{j}\log\left(\hat{y_{j}}\right) \end{array}$$

Where *y*_*j*_ is the true label and $\hat{y_{j}}$ is the predicted probability for the *j*-th class.

### 4.5 Acoustic data: Long Short-Term Memory (LSTM) network

The acoustic data consists of bird calls and songs from the *iBC53 dataset*, which includes recordings from diverse Indian habitats.

#### 4.5.1 Preprocessing steps

Audio recordings were cleaned using spectral subtraction to reduce background noise. *Mel-Frequency Cepstral Coefficients (MFCCs)* were extracted from the audio samples, representing the timbre and pitch of the bird calls. The MFCCs are computed as:


(21) $$\begin{array}{l} c_{m}=\overset{N}{\underset{n=1}{\sum }}X_{n}\cos\left[\frac{m\left(n-0.5\right)\pi }{N}\right] \end{array}$$

Where *X*_*n*_ is the magnitude of the signal at frame *n*, and *m* is the MFCC coefficient.

#### 4.5.2 LSTM architecture

The LSTM network processes the MFCC features by utilizing memory cells, which operate according to the following set of equations:


(22) $$\begin{array}{l} f1_{t}=\sigma \left(W_{f}\cdot \left[h_{t-1},x_{t}\right]+b_{f}\right) \end{array}$$

(23) $$\begin{array}{l} i1_{t}=\sigma \left(W_{i}\cdot \left[h_{t-1},x_{t}\right]+b_{i}\right) \end{array}$$

(24) $$\begin{array}{l} {\tilde{C1}}_{t}=\tanh\left(W_{C}\cdot \left[h_{t-1},x_{t}\right]+b_{C}\right) \end{array}$$

(25) $$\begin{array}{l} C1_{t}=f1_{t}*C_{t-1}+i1_{t}*{\tilde{C1}}_{t} \end{array}$$

(26) $$\begin{array}{l} o1_{t}=\sigma \left(W_{o}\cdot \left[h_{t-1},x_{t}\right]+b_{o}\right) \end{array}$$

(27) $$\begin{array}{l} h1_{t}=o1_{t}*\tanh\left(C1_{t}\right) \end{array}$$

In this structure, *f*1_*t*_ refers to the forget gate, *i*1_*t*_ is the input gate, and *o*1_*t*_ represents the output gate. The cell state is denoted by *C*1_*t*_, which is updated by combining the previous state and new candidate information based on the operations of the forget and input gates.

This architecture enables the LSTM to efficiently retain important information over extended sequences, mitigating issues that arise in standard RNNs like the vanishing gradient problem.

#### 4.5.3 Training

The LSTM model was trained using *RMSprop optimizer* with *categorical cross-entropy loss*. A fixed learning rate of 0.001 and batch size of 64 were used.

Table 6 compares the deep learning architectures applied to visual and acoustic data. For the visual modality (ResNet-50), there are 49 convolutional layers, 2 pooling layers (1 max pooling and 1 global average pooling), and 1 fully connected layer, resulting in a feature vector size of 2048. In contrast, the acoustic modality, based on LSTM networks, does not include convolutional layers as MFCC features are directly extracted from the audio input. It has no pooling layers and 1 fully connected layer after the LSTM, producing feature vectors of size 128 or 256. Finally, the fused modality combines the visual and acoustic features into a single fully connected fusion layer, producing a feature vector size of 2304, capturing the integrated information from both data sources."

**Table 6:** Summary of layers.

**Modality**	**Convolutional layers**	**Pooling layers**	**Fully connected layers**	**Feature vector size**
Visual (ResNet-50)	49 layers	2 layers (1 max, 1 global avg)	1 (dense)	2,048
Acoustic (LSTM)	0 (MFCC is extracted, no CNN used here)	None	1 (after LSTM)	128 or 256
Fused layer	N/A	N/A	1 (for fusion)	2,304

### 4.6 Multimodal fusion

We explored two primary strategies for integrating visual and acoustic modalities in species identification: *early fusion* and *late fusion*. Both strategies leverage the strengths of each modality to enhance classification performance.

#### 4.6.1 Components of hybrid fusion

##### 4.6.1.1 Feature extraction

- **Visual features**: advanced deep learning models, particularly Deep Convolutional Neural Networks (DCNNs), are employed to extract intricate visual features from bird images. These features encompass aspects like shape, color patterns, and other distinguishing visual characteristics.
- **Acoustic features**: to analyze audio data, models such as Long Short-Term Memory (LSTM) networks are utilized. Acoustic features are extracted through techniques like Mel-frequency cepstral coefficients (MFCCs), which capture the spectral properties of bird calls and other audio patterns relevant to species identification.

##### 4.6.1.2 Fusion strategies

Early and late fusion techniques are methods used to combine visual and acoustic information for bird species identification. In early fusion, features from both types of data–such as images and sounds–are merged early in the process, allowing the model to analyze them together and learn interactions between the two. This approach helps the model to utilize complementary information from both modalities simultaneously. Late fusion, on the other hand, involves processing visual and acoustic data separately through their respective models and then combining their predictions at the end, using methods like weighted averages. While early fusion emphasizes understanding the relationships between the two data types during training, late fusion focuses on combining their individual strengths after independent analysis.

- **Early fusion**: In this approach, visual and acoustic features are concatenated into a single feature vector before being fed into the classifier. This allows the model to jointly learn from both modalities simultaneously.
- **Late Fusion**: Here, separate classifiers are trained for each modality (visual and acoustic), and their respective outputs are fused post-classification, typically through a weighted combination of their predictions.

#### 4.6.2 How Hybrid Fusion Works

##### 4.6.2.1 Feature extraction process

- Visual data is processed through a DCNN to extract features, resulting in a feature vector *f*_visual_.
- Acoustic data is preprocessed, and relevant features are extracted, generating a feature vector *f*_acoustic_.

##### 4.6.2.2 Early fusion implementation


(28) $$\begin{array}{l} f_{\text{fused\_early}}=\left[f_{\text{visual}},f_{\text{acoustic}}\right] \end{array}$$

The visual and acoustic features are concatenated into a single vector. This fused feature vector is then passed through a fully connected neural network for classification, allowing the model to learn from both modalities in tandem.

##### 4.6.2.3 Late fusion implementation


(29) $$\begin{array}{l} p_{\text{final\_late}}=\alpha p_{\text{visual}}+\left(1-\alpha \right)p_{\text{acoustic}} \end{array}$$

Separate classifiers are trained for the visual and acoustic features, producing probabilities *p*_visual_ and *p*_acoustic_ for each modality. The final prediction *p*_final_late_ is obtained by combining the outputs through a weighted sum, where α is a hyperparameter that is optimized during the validation phase.

**Figure 2:**
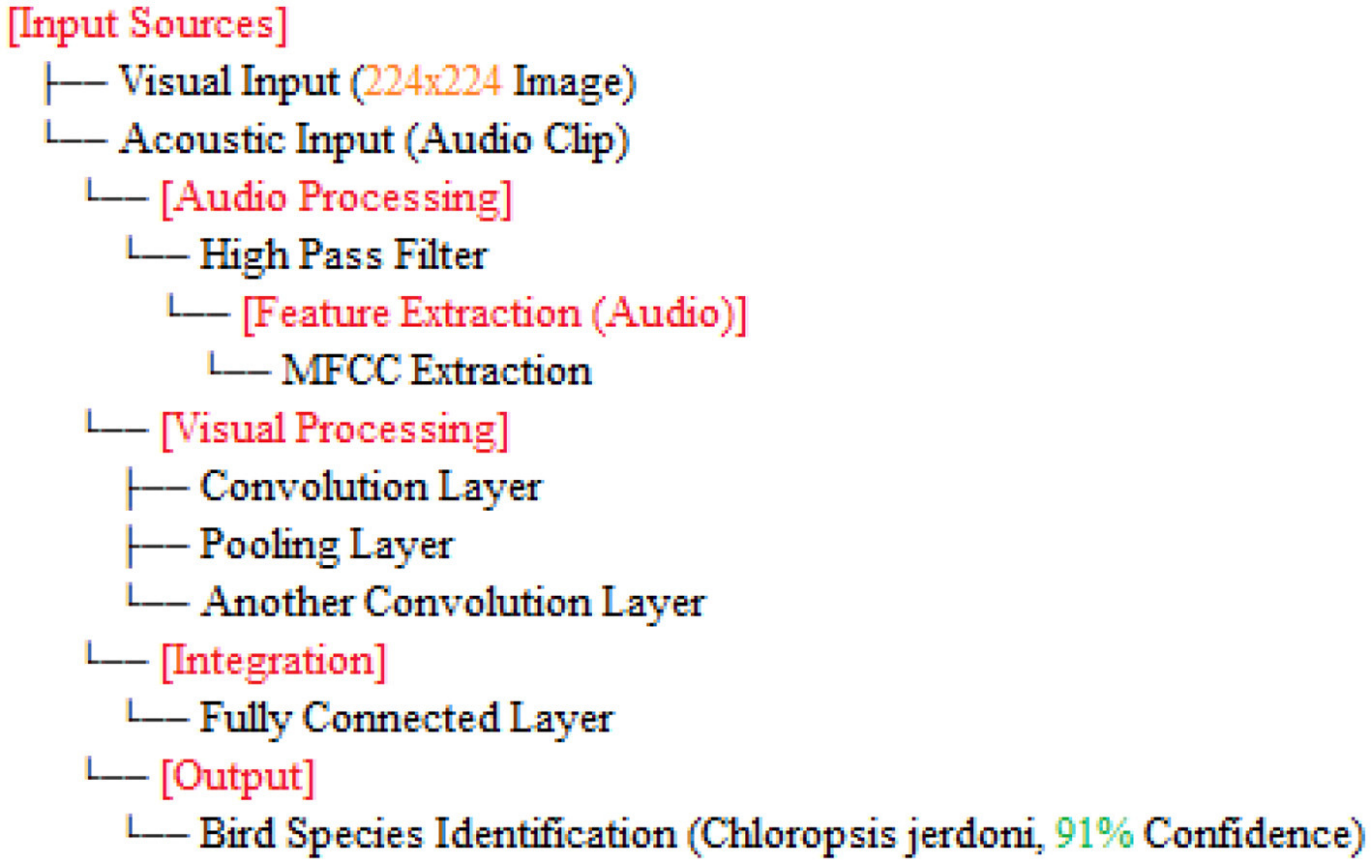
Steps design for bird species identification using visual and acoustic inputs (Chloropsis_jerdoni).

The algorithm 1 classifies Chloropsis jerdoni using a multimodal approach by integrating image, audio, and location data. First, the input data is preprocessed–images are normalized, audio is converted into spectrograms, and location metadata is standardized. Features are then extracted from each modality: a CNN is used for image features, an RNN or transformer model for audio spectrograms, and a dense network for location data. These features are fused into a single vector, optionally reduced in dimensionality. A multimodal deep learning model is trained on these fused features using a suitable loss function and optimizer. For testing, the model processes the features from unseen samples to predict class probabilities, identifying Chloropsis jerdoni if the probability exceeds a defined threshold. Finally, the model's performance is validated using metrics such as accuracy, precision, recall, F1-score, and AUC-ROC, ensuring reliable classification. the overall step by step explaination is shown in Figure 2.

**Algorithm 1:** Bird Classification for Chloropsis jerdoni bird using a multimodal approach.

1: **Input:** Bird images *I* = {*I*_1_, *I*_2_, …, *I*_*n*_}, audio file *A*
2: **Output:** Classification results *C*
3: **Step 1: Data Collection**
4: Acquire bird images *I* and audio file *A*
5: **Step 2: Preprocessing**
6: Resize images: *I*_*i*_←Resize(*I*_*i*_, target_size)
7: Apply data augmentation: $I_{i}^{\prime }\leftarrow \text{Augment}\left(I_{i}\right)$
8: Reduce noise in audio: *A*′←Denoise(*A*)
9: Extract MFCCs: *MFCC* = MFCC(*A*′)
10: Encode labels: *y*_*i*_∈{0, 1, …, *k*−1} for *k* classes
11: **Step 3: Feature Extraction**
12: Extract visual features: *V* = ResNet-50(*I*)
13: Extract acoustic features: *A*_*features*_ = LSTM(*MFCC*)
14: **Step 4: Multimodal Fusion**
15: Early Fusion: *F*_*early*_←*V*⊕*A*_*features*_ // Concatenate visual and acoustic features
16: Late Fusion: Train separate classifiers *C*_*V*_ (visual) and *C*_*A*_ (acoustic)
17: Combine outputs: *C*←Fusion(*C*_*V*_, *C*_*A*_)
18: **Step 5: Model Training**
19: Train model: θ ← Train(*F*_*early*_, *y*) using Adam optimizer
20: Optimize with categorical cross-entropy loss:
$L\left(\theta \right)=-\overset{n}{\underset{i=1}{\sum }}y_{i}\log\left(\hat{y_{i}}\right)$
21: **Return:** Classification results *C*

#### 4.6.3 Benefits of hybrid fusion

- **Enhanced classification accuracy**: by combining both fusion strategies, the system benefits from the strengths of each, leading to more accurate species classification.
- **Robustness to noise**: the independent processing of visual and acoustic modalities helps mitigate the impact of noise in one modality, improving the system's overall robustness.
- **Comprehensive feature representation**: hybrid fusion allows the model to capture complex relationships between modalities while maintaining the uniqueness of the features from each source.

This approach significantly advances ecological monitoring and species identification, leveraging visual and acoustic data for more accurate and reliable classification. The model offers a comprehensive solution for identifying bird species in diverse environments by effectively integrating multimodal inputs.

## 5 Experimental results

### 5.1 Dataset and experimental setup

The models were trained and evaluated on the *iBC53* dataset, which contains 10,000 images and 5,000 audio recordings representing 53 bird species from across India. The dataset was split into 70% training, 15% validation, and 15% test sets.

This section presents the experimental evaluation of the proposed *Visual-acoustic fusion techniques* for Indian bird species identification. We assess the model performance using both visual and acoustic data, and further analyze the improvements brought by multimodal fusion techniques in terms of classification accuracy, robustness, and modality contributions.

For the visual modality, we fine-tuned a pre-trained Inception-ResNet-152 model using bird images, extracting deep visual features. For the acoustic modality, audio features were extracted using Mel-frequency cepstral coefficients (MFCCs), and a Long Short-Term Memory (LSTM) network was employed to analyze sequential audio data. The two fusion techniques, early and late fusion, were implemented and tested to evaluate their effectiveness.

### 5.2 Results for visual and acoustic modality alone

To establish baseline results, we first evaluated the performance of individual modalities shown in Table 7. The fine-tuned visual model (DCNN) achieved an accuracy of 87.2%, while the acoustic model (LSTM with MFCCs) attained an accuracy of 84.3%. These results highlight the classification potential of each modality, though both exhibit certain limitations when used independently.

**Table 7:** Performance of individual modalities.

**Modality**	**Accuracy**	**F1-score**
Visual modality (DCNN)	87.2%	0.85
Acoustic modality (LSTM with MFCC)	84.3%	0.82

### 5.3 Performance comparison: early fusion vs. late fusion

**Figure 3:**
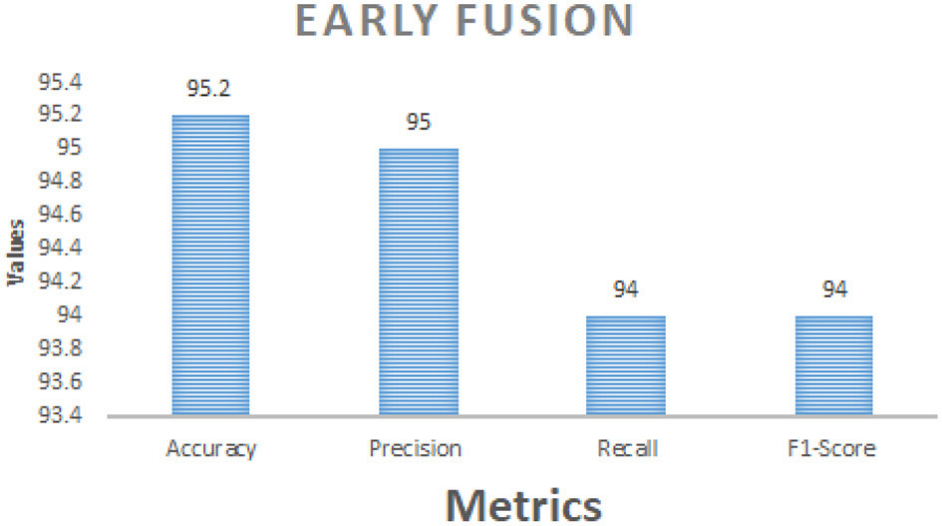
Performance metrics of early fusion strategy.

**Figure 4:**
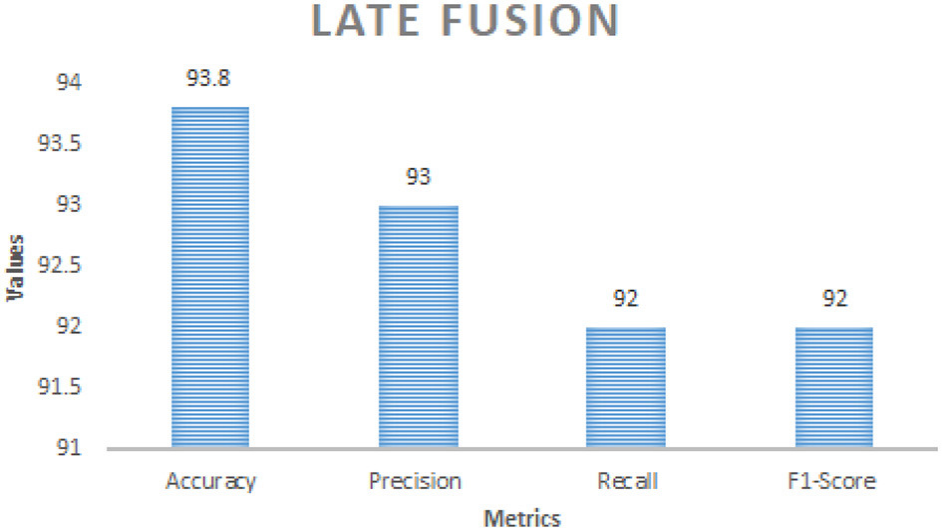
Performance metrics of late fusion strategy.

Table 8 comprises Performance comparison of early fusion and late fusion approaches for multi modal bird species identification. In the early fusion approach, visual and acoustic features are concatenated into a single feature vector before classification, leading to a significant accuracy improvement of 95.2%. This method allows the model to capture complementary features from both modalities, resulting in superior classification performance shown in the Figure 3. In contrast, the late fusion approach trains independent classifiers for visual and acoustic data, with their final predictions combined using a weighted sum. This method achieved an accuracy of 93.8%, demonstrating slightly lower performance than early fusion but offering greater robustness in situations with noisy or incomplete data which is shown in the Figure 4. The comparison underscores the strengths of each fusion strategy, with early fusion excelling in accuracy and late fusion providing greater resilience to data imperfections.

**Table 8:** Performance comparison between early fusion and late fusion techniques for bird species identification.

**Metric**	**Early fusion**	**Late fusion**
Accuracy	95.2%	93.8%
Precision	95%	93%
Recall	94%	92%
F1-Score	94%	92%

### 5.4 Comparison of fusion techniques

**Figure 5:**
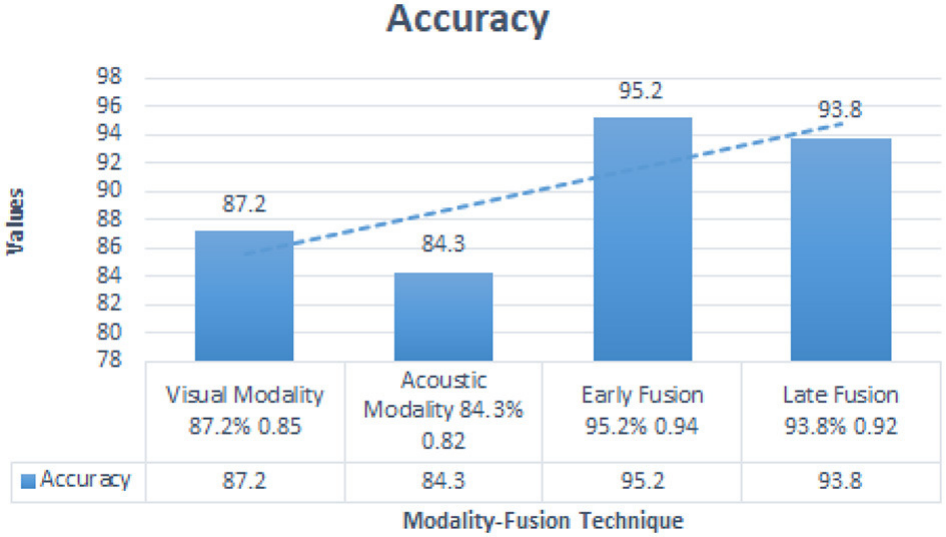
Comparison of accuracy across fusion techniques.

The fusion techniques consistently outperformed individual modality classifiers. Early fusion, with an accuracy of 95.2%, was superior when both modalities were reliable. Late fusion, with an accuracy of 93.8%, proved more robust when one modality was noisy or missing, offering an effective backup in cases of incomplete data. Table 9 below provides a comparative summary of visual modality, acoustic modality, early fusion and late fusion which is shown in Figure 5.

**Table 9:** Performance comparison of fusion techniques.

**Method**	**Accuracy**	**F1-score**
Visual Modality	87.2%	0.85
Acoustic Modality	84.3%	0.82
Early Fusion	95.2%	0.94
Late Fusion	93.8%	0.92

### 5.5 Robustness to noise and missing data

**Figure 6:**
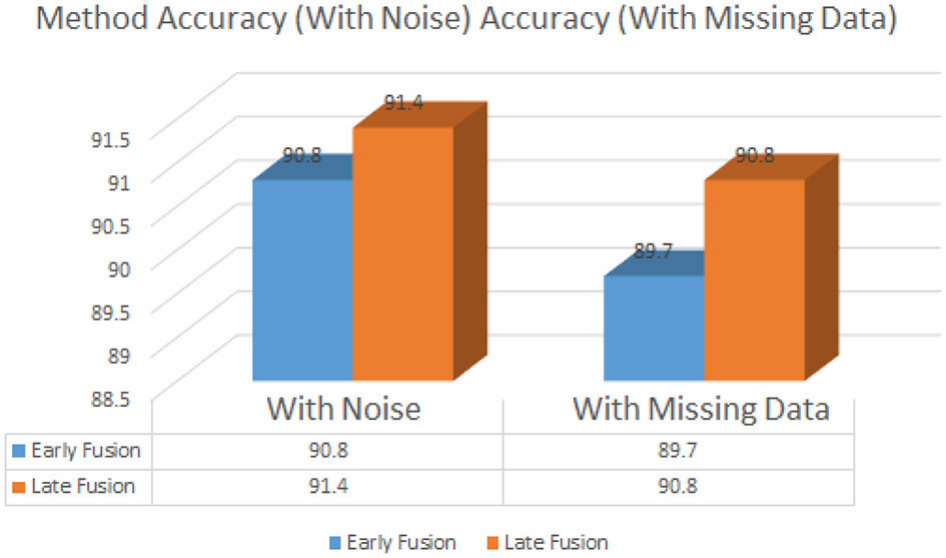
Accuracy under noisy and missing data conditions.

Table 10 evaluated the robustness of both fusion strategies by introducing noise into the audio recordings and removing visual data from a subset of samples. The late fusion strategy exhibited greater resilience, with accuracy dropping only slightly from 93.8% to 91.4%. Early fusion, though more accurate under normal conditions, experienced a more pronounced drop, from 95.2% to 90.8%, when confronted with incomplete data or noisy inputs.the detailed accuracy comparison is shown in the Figure 6.

**Table 10:** Robustness to noise and missing data.

**Method**	**Accuracy (with noise)**	**Accuracy (with missing data)**
Early fusion	90.8%	89.7%
Late fusion	91.4%	90.8%

## 6 Discussion

The experimental results validate the advantages of multi modal fusion in bird species identification tasks. Both fusion techniques demonstrated substantial improvements over using visual or acoustic data alone. Early fusion yielded the highest accuracy (95.2%), making it ideal for scenarios where data from both modalities are consistently available and of high quality. Late fusion, while slightly less accurate, offered enhanced robustness in real-world conditions where noise or incomplete data are more likely.

Both fusion techniques achieved high accuracy, and the early fusion yielded slightly better results because of the effective integration of complementary visual and acoustic information. The exact accuracy values for both approaches are reported in the results section, where the early fusion model shows a significant improvement in species identification, particularly in challenging environments with noisy backgrounds or incomplete data.

These results underscore the effectiveness of multi modal fusion for ecological monitoring, where data from multiple modalities can be harnessed for improved performance. Future research could explore adaptive fusion techniques that dynamically adjust to varying data quality, further optimizing system performance in challenging environments.

The accuracy in this study is determined by evaluating the percentage of correctly identified bird species across the test dataset. For both early and late fusion techniques, the model's predictions are compared with the true labels in the iBC53 dataset. The accuracy metric considers all correctly classified species (both visual and acoustic inputs) out of the total samples. When evaluating the multimodal fusion approaches, the parameter includes the combined contributions of visual and acoustic modalities, allowing for a direct comparison of the effectiveness of early versus late fusion strategies. Additional metrics such as precision, recall, and F1-score complement the accuracy measure to provide a more comprehensive evaluation of the model's performance. The performance of the proposed model was measured using various parameters, including accuracy, precision, recall, F1-score, and AUC-ROC. These metrics were evaluated for both early and late fusion techniques, with early fusion consistently outperforming late fusion across all parameters.

**Figure 7:**
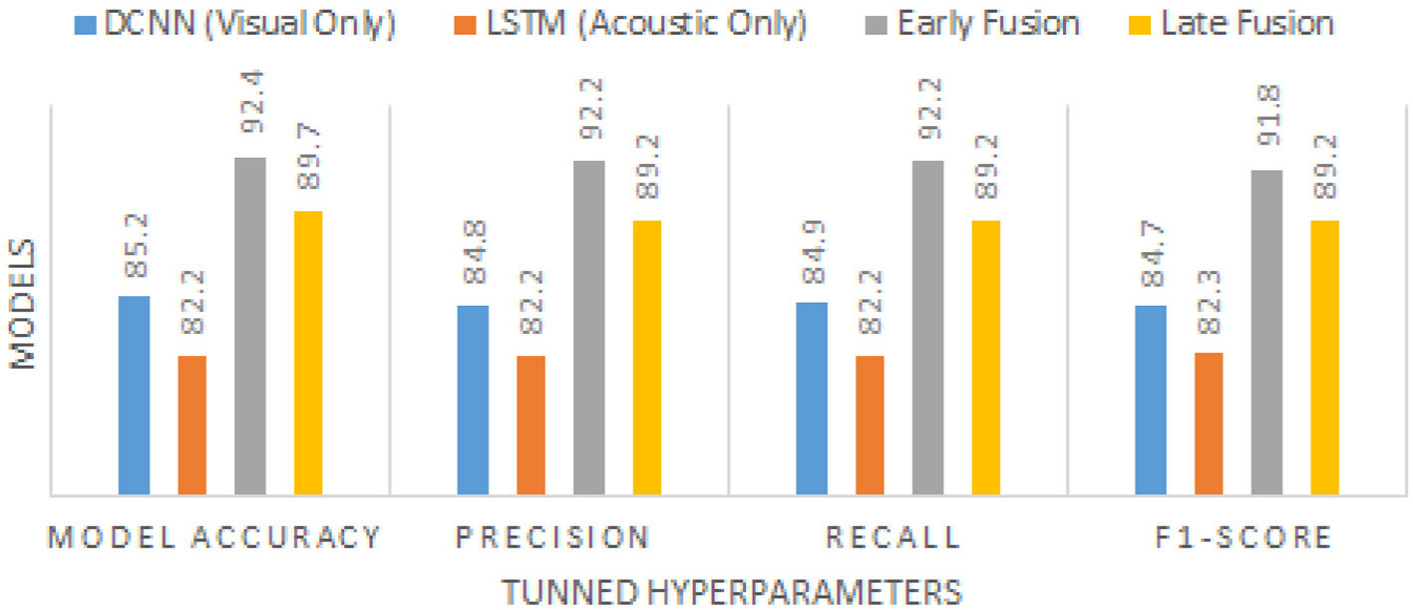
Performance comparison of different models.

The models were evaluated using *accuracy, precision, recall*, and *F1-score*. Early fusion outperformed both individual modalities and late fusion, as shown in Table 11 with the performance graph illustrated in Figure 7.

**Table 11:** Performance comparison of different models.

**Model**	**Accuracy**	**Precision**	**Recall**	**F1-score**
DCNN (visual only)	85.2%	84.8%	84.9%	84.7%
LSTM (acoustic only)	82.5%	82.2%	82.1%	82.3%
Early fusion	**92.4%**	**92.2%**	**92.0%**	**91.8%**
Late fusion	89.7%	89.2%	89.3%	89.2%

The bold values represent the highest (or most significant) values within each category, highlighting key findings or optimal results.

## 7 Conclusion

A multi-modal framework for identifying bird species was introduced, utilizing deep learning techniques to combine visual and audio data. The method demonstrated exceptional performance through the use of early fusion techniques, making it particularly beneficial for ecological research and biodiversity preservation in India. The findings highlight that accuracy and robustness are significantly enhanced by combining the complementary strengths of DCNNs and LSTMs, offering a reliable approach to addressing the challenges of multi-modal data and environmental variability.

### 7.1 Future work

Future work will focus on exploring adaptive fusion strategies that dynamically adjust modality weights based on data quality. In addition, our goal is to incorporate environmental data as an additional modality and develop real-time bird species identification capabilities for broader applications, including conservation efforts and citizen science projects.

## Data Availability

The datasets presented in this study can be found in online repositories. The names of the repository/repositories and accession number(s) can be found below: https://www.kaggle.com/datasets/arghyasahoo/ibc53-indian-bird-call-dataset.
